# Activation of Anionic Redox for Stoichiometric and Li-Excess Metal Sulfides through Structural Disordering: Joint Experimental and Theoretical Study

**DOI:** 10.1021/jacs.5c04018

**Published:** 2025-07-15

**Authors:** Miyuki Shinoda, Koki Matsunoshita, Masanobu Nakayama, Satoshi Hiroi, Koji Ohara, Masaki Abe, Nozomu Ishiguro, Yukio Takahashi, Gen Hasegawa, Naoaki Kuwata, Tsukasa Iwama, Takuya Masuda, Kosuke Suzuki, Hirofumi Ishii, Yu-Cheng Shao, Daisuke Shibata, Akinori Irizawa, Toshiaki Ohta, Itsuki Konuma, Teppei Ohno, Yosuke Ugata, Naoaki Yabuuchi

**Affiliations:** † Department of Chemistry and Life Science, 13154 Yokohama National University, 79-5 Tokiwadai, Hodogaya-ku, Yokohama, Kanagawa 240-8501, Japan; ‡ Department of Advanced Ceramics, 12982 Nagoya Institute of Technology, Gokiso-cho, Showa-ku, Nagoya, Aichi 466-8555, Japan; § Faculty of Materials for Energy, 12938 Shimane University, 1060 Nishikawatsu-cho, Matsue, Shimane 690-8504, Japan; ∥ RIKEN SPring-8 Center, 1-1-1, Koto, Sayo, Hyogo 679-5148, Japan; ⊥ Research Center for Energy and Environmental Materials (GREEN), 52747 National Institute for Materials Science (NIMS), 1-1 Namiki, Tsukuba, Ibaraki 305-0044, Japan; # International Center for Synchrotron Radiation Innovation Smart (SRIS), 13101 Tohoku University, 2-1-1 Katahira, Aoba-ku, Sendai, Miyagi 980-8577, Japan; ∇ Department of Metallurgy, Materials Science and Materials Processing, Graduate School of Engineering, Tohoku University, 6-6-2 Aoba-yama, Aoba-ku, Sendai, Miyagi 980-8579, Japan; ○ Institute of Multidisciplinary Research for Advanced Materials (IMRAM), Tohoku University, 2-1-1 Katahira, Aoba-ku, Sendai, Miyagi 980-8577, Japan; ◆ Institute for Materials Research, Tohoku University, 2-1-1 Katahira, Aoba-ku, Sendai, Miyagi 980-8577, Japan; ¶ Graduate School of Chemical Sciences and Engineering, Hokkaido University, Sapporo, Hokkaido 060-0810, Japan; †† Graduate School of Science and Technology, Gunma University, 1-5-1 Tenjin-cho, Kiryu, Gunma 376-8515, Japan; ‡‡ 57815 National Synchrotron Radiation Research Center, Hsinchu 30076, Taiwan; §§ SR Center, 12696 Ritsumeikan University, Kusatsu, 1-1-1 Nojihigashi, Kusatsu, Shiga 525-8577, Japan; ∥∥ Advanced Chemical Energy Research Center, Institute of Advanced Sciences, Yokohama National University, 79-5 Tokiwadai, Hodogaya-ku, Yokohama, Kanagawa 240-8501, Japan

## Abstract

Extensive research efforts have been dedicated to Li-excess compounds with anionic redox reaction as potential high-capacity positive electrode materials for Li-ion battery applications. The origin of activation on anionic redox is still under debate, and a unified understanding is necessary, especially for sulfide-based compounds without conductive d electrons. Herein, joint experimental and theoretical study is conducted for Li-excess and stoichiometric compounds with different crystal structures, cation-disordered rocksalt, and cation-ordered layered structures. In contrast to the understanding of Li-excess oxides, sulfide-based compounds with ordered layered structures are electrochemically less active compared with the materials with disordered structure. Theoretical study reveals that a unique local structure for a sulfide ion coordinated by 6 Li ions, SLi_6_ configuration, is formed, which can be found only for the disordered structure and not for the layered structure. The unique local structure triggers electron delocalization for sulfide 3p orbitals, leading to superior electronic conductivity, as experimentally evidenced, and thus anionic redox is successfully activated. Furthermore, such nonuniform local structures lead to easier structural distortion and efficient S–S dimerization, and even trimerization (S_3_^2–^), upon delithiation. Although sulfide-based compounds as battery electrode materials suffer from dissolution after oxidation, this practical problem is effectively mitigated by the use of highly concentrated electrolyte solutions with fewer free solvent molecules, leading to superior reversibility for anionic redox. The insights derived can guide the development of high-energy electrode materials with anionic redox with or without transition metal ions possessing conductive d electrons.

## Graphical abstract

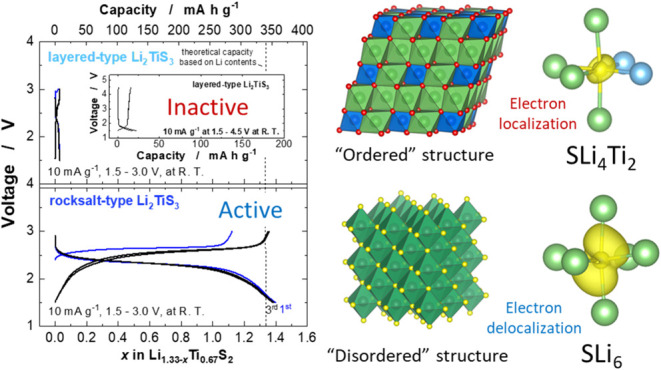

## Introduction

Technological progress on rechargeable batteries is indispensable toward sustainable energy development and fossil fuel-free society. The research achievements on rechargeable batteries successfully resulted in the electrification of automobiles, and the market of electric vehicles is rapidly growing in the world. Currently, Ni-enriched layered materials, LiNi_1–2*x*_Co_*x*_Mn_*x*_O_2_, are widely used for batteries in state-of-the-art electric vehicles. Reversible capacities of 170–220 mA h g^–1^ with excellent cyclability are obtained using redox reaction of cationic species, Ni^2+^/Ni^3+^/Ni^4+^, Co^3+^/Co^4+^.^1,2^ Recently, the electrode reversibility of Co-free LiNiO_2_ has been effectively improved through the engineering of structural defects coupled with surface stabilization.^3,4^ Nevertheless, cost-effective and Ni-/Co-free electrode materials are necessary to further accelerate this movement in automobile applications. Lithium-excess and manganese-based electrode materials, Li_2_MnO_3_ (Li_4/3_Mn_2/3_O_2_) and its derivatives, were extensively studied in the past decade. The enrichment of Li-ion fractions in the host structure leads to higher theoretical capacity as electrode materials. Large reversible capacities are delivered for these electrode materials through the activation of anionic redox reaction, namely, charge compensation by oxide ions on oxidation.^5–8^ The emerging new chemistry, anionic redox, potentially boosts the energy density of Li-ion batteries. Nevertheless, the reversibility of anionic redox for oxide ions is not high enough associated with gradual oxygen loss for continuous cycles and thus hinders its use for practical applications.

Recently, this concept of anionic redox for Li-excess electrode materials is extended to sulfide ions, which are soft and easily polarizable ions compared with hard oxide ions. Historically, Li_2_FeS_2_ was studied in 1987 as electrode material and recently revisited as a model electrode material. Both cationic (Fe^2+^/Fe^3+^) and anionic (2S^2–^/S_2_^2–^) redox are activated in Li_2–*x*_FeS_2_, and reversible dimer formation of sulfur species proceeds on electrochemical oxidation and lithium removal.^9^ Recently, Li_2_TiS_3_ polymorphs and its derivatives have also been proposed as a new series of Li-excess sulfide system.^10–13^ Li_2_TiS_3_ polymorphs show the unique electrode performance, and Li_2_TiS_3_ with the cation-disordered rocksalt structure delivers a high-reversible capacity of over 300 mA h g^–1^ by using sulfur-based anionic redox coupled with the percolative Li-ion migration. In contrast, surprisingly, Li_2_TiS_3_ with the layered structure is electrochemically inactive.^12^ Ordered Li_3_NbS_4_ is also inactive as electrode materials, but a disordered phase shows good electrode reversibility.^14^ Similarly, LiMn_0.5_Ti_0.5_S_2_ with the rocksalt structure shows better performance when compared with the sample with the layered structure.^15^ This trend, better reversibility for samples with the rocksalt structure, is clearly different from that of LiCoO_2_, which is the most widely used layered oxide for battery applications. Cation-ordered and layered LiCoO_2_ shows excellent electrode reversibility, while cation-disordered LiCoO_2_ shows inferior performance as electrode materials.^16^ Electrode reversibility of layered Li_2_TiS_3_ is also activated when solid solution phases are formed, for instance, Li_2_TiS_3_–LiTiS_2_ and Li_2_TiS_3_–LiFe_0.5_Ti_0.5_S_2_ binary systems.^12^ Anion substitution is also proposed to activate electrode reversibility, *i.e*., Li_2_TiS_3–*x*_Se_*x*_.^17^ Nevertheless, the origin of activation of the anionic redox for Li-excess sulfide-based materials is under debate. For battery applications, many sulfides are studied for solid electrolyte and these compounds are designed with main-group elements, e.g., Li_3_PS_4_, Li_10_GeP_2_S_12_, Li_6_PS_5_Cl, *etc*. Transition metal ions are eliminated from these compounds used for solid electrolytes because transition metal ions contain conductive d-electrons. The solid electrolyte is ideally required to be a pure ionic conductor and an electronic insulator. In addition, sulfide ions have a reductive nature, and transition metal ions with higher oxidation states are easily reduced by sulfide ions. Therefore, only limited transition metal ions are used for the material synthesis, and there are relatively few studies on sulfide-based compounds with transition metal ions.

In this study, systematic studies on Li_2_TiS_3_ with different structures are conducted through joint experimental and theoretical approaches. For comparison, non-Li-excess and stoichiometric system, *i.e*., rocksalt LiMn_1/2_Ti_1/2_S_2_,^15^ is also synthesized and characterized, and these results are compared with those of Li_2_TiS_3_ (Li_4/3_Ti_2/3_S_2_). These electrode materials deliver large reversible capacity, which nearly corresponds to theoretical capacities based on Li contents with solely anionic redox, which cannot be achieved for oxide-based layered compounds. From these results, the factors affecting sulfur-based anionic redox and electrode reversibility are discussed, through which the feasibility of Li-excess metal sulfides for potential battery applications is also evaluated.

## Experimental Section

### Synthesis of Materials

Li_2_TiS_3_ and LiMn_0.5_Ti_0.5_S_2_ with a disordered structure were synthesized from Li_2_S (Sigma-Aldrich), TiS_2_ (Sigma-Aldrich), and MnS (Sigma-Aldrich) by mechanical milling using a planetary ball mill (PULVERISETTE 7; FRITSCH) with a ZrO_2_ pot (45 mL) and balls at 600 rpm for 12 h. The samples were handled in an Ar-filled glovebox to avoid contact with moisture. Structural evolutions of the samples by milling were studied using an X-ray diffractometer (D2 PHASER, Bruker) equipped with a high-speed one-dimensional detector using Cu Kα radiation generated at 300 W (30 kV and 10 mA) with a Ni filter.

### Electrochemical Characterization

Composite positive electrodes composed of 80 wt % Li_2_TiS_3_ or LiMn_0.5_Ti_0.5_S_2_, 10 wt % acetylene black, and 10 wt % polyvinylidene fluoride (KF 1100; Kureha Co. Ltd.) were dispersed in *N*-methylpyrrolidone and pasted on aluminum foil as a current collector. The electrodes were dried at 120 °C for 2 h in a vacuum. Metallic lithium (Honjo Metal Co., Ltd.) was used as a negative electrode. The electrolyte solution used was 1.0 mol dm^–3^ LiPF_6_ dissolved in ethylene carbonate/dimethyl carbonate (3:7 by volume, battery grade; Kishida Chemical Co. Ltd.) and 7.25 mol kg^–1^ LiBF_4_ dissolved in propylene carbonate (battery grade; Kishida Chemical Co. Ltd). A porous polyolefin membrane was used as a separator for the LiPF_6_ electrolyte, and a glass filter (GB-100R, Advantec) was used as a separator for 7.25 mol kg^–1^ LiBF_4_ electrolyte with high viscosity. Two-electrode cells (TJ-AC; Tomcell) were assembled in an Ar-filled glovebox.

### Materials Characterization

Particle morphology of the samples was observed by using a scanning electron microscope equipped with an energy dispersive X-ray spectrometer (SEM-EDS) (JCM-6000, JEOL). *In situ* XRD data were collected with a battery cell equipped with a Be window combined with an X-ray diffractometer (D8 ADVANCE; Bruker Corp., Ltd.). Soft X-ray absorption spectroscopy (XAS) data were collected at BL-13 (S K-edge and Ti K-edge) in the synchrotron facility of Ritsumeikan University (Synchrotron Radiation Center). Absorption spectra were acquired using the fluorescence yield mode. Hard XAS spectra at the Mn K-edge were collected at beamline BL-12C of the Photon Factory Synchrotron Source in Japan. X-ray total scattering measurements were performed at two beamlines in SPring-8, Japan, to study the local structure by pair distribution function (PDF) analysis. An incident X-ray energy of *E* = 61.3 keV was used at BL04B2 beamline with hybrid detectors of Ge and CdTe. Measurements were conducted using a two-dimensional CdTe detector (Eiger2 4M) with an incident X-ray energy of 67.0 keV at the BL12B2 beamline. The reduced PDF *G*(*r*) was obtained by the conventional Fourier transform of *S*(*Q*).^18^ The local structure around Li was evaluated by ^7^Li magic angle spinning nuclear magnetic resonance (MAS NMR) using an ECA-400 spectrometer (JEOL Ltd., Japan). The resonance frequency of the ^7^Li nucleus was 155.4 MHz. A 1.0 mol dm^–3^ LiCl aqueous solution was used as the chemical shift reference at 0 ppm. A 3.2 mm MAS probe (HXMAS probe; JEOL) and a 3.2 mm zirconia sample tube were used. The MAS spinning rate was varied between 0 and 20 kHz. The width of the π/2 pulse was 2.8 μs. The pulse-recycling period was kept longer than 17 s to confirm the spin recovering. The NMR spectra were obtained by an MAS-rotation synchronized spin–echo pulse sequence. Hard X-ray photoelectron spectroscopy (HAXPES) measurements were carried out using a custom-made system equipped with a Cr Kα (5414.9 eV) focused X-ray source (Ulvac-Phi) operating at 50 W and an EW-4000 electron analyzer (Scienta Omicron). The incident angle of X-rays and takeoff angle of photoelectrons were fixed at 75 and 75°, respectively. The energy of the photoelectrons passing through the analyzer (pass energy) was fixed at 200 eV. The vacuum pressure in the analysis chamber was ∼2 × 10^–9^ mbar. This system was directly attached to an Ar-filled glovebox, so that the samples can be directly transferred to the analysis chamber without exposure to open air. Magnetization measurements for Li_2–*x*_TiS_3_ were performed by using a superconducting quantum interference device (SQUID) magnetometer (MPMS5-SW, Quantum Design, Inc.). The magnetizations for all samples were measured by changing the sample temperature from 300 to 12 K under a constant magnetic field of 1000 Oe.

### Density Functional Theory Calculations

First-principles calculations based on density functional theory (DFT) for Li_2_(1–*x*)­TiS_3_ system were performed using Vienna Ab initio Simulation Package (VASP)^19,20^ with modified Perdew–Burke–Ernzerhof generalized gradient approximation (PBEsol-GGA)^21,22^ and with the projector-augmented wave (PAW) method.^23^ Cutoff was set as 500 eV, and the *k*-point meshes were sampled such that the product of the meshes and atoms was approximately 600.

## Results and Discussion

### Synthesis and Characterization of Rocksalt and Layered Li_2_TiS_3_

Li-excess titanium sulfide, Li_2_TiS_3_, was synthesized from a mixture of Li_2_S and TiS_2_ by high-energy mechanical milling. X-ray diffraction (XRD) patterns of the samples before and after milling are compared in Supporting Figure S1. After mechanical milling, diffraction lines for starting materials disappear and a low-crystallinity sample with cation-disordered rocksalt sulfide is formed. Nanosized materials synthesized by mechanical milling generally consist of agglomerated nanosized grains (∼10 nm size) with enriched grain boundaries.^24,25^ This rocksalt sulfide is a metastable phase, and after heat treatment at 500 °C for 3 h, a layered sulfide with high crystallinity is obtained. Structural evolution from the rocksalt to layered phase on heating is observed in Figure 1a. The metastable rocksalt phase is stable after heating at 300 °C. Partial crystallization of the layered phase is evidenced after heating at 400 °C, and a small 001 diffraction line is observed for the sample heated at 400 °C. The layered phase with high crystallinity is immediately formed after heating at 500 °C. A superlattice line is also observed at 16.5°, indicating the presence of honeycomb-type in-plane cation ordering for Li and Ti ions. The particle morphology of the sample observed by using a scanning electron microscope (SEM) is also shown in Figure 1b. The increase in particle size after heat treatment is found in SEM images, and Brunauer–Emmett–Teller (BET) measurement reveals that the specific surface area is reduced from 18.1 to 4.3 m^2^ g^–1^ after heat treatment. A similar trend, crystallization of a layered phase from disordered rocksalt phase by heating, is observed in stoichiometric LiMn_0.5_Ti_0.5_S_2_ as reported in the literature.^15^ Local structures of rocksalt and layered Li_2_TiS_3_ have been compared with total X-ray scattering with pair distribution function (PDF) analysis as shown in Figure 1c. A clear difference in the first coordination shells for both samples is noted. Lower peak intensity is observed for rocksalt Li_2_TiS_3_, which is indicative of a local distortion for the nanosized sulfide with enriched grain boundaries. In addition, a peak intensity difference is more clearly visible as the *r* value increases, indicating that long-range ordering is more significant for the layered phase as expected from the difference in crystallinity for both samples. These differences in local structures are further evidenced from ^7^Li magic angle spinning nuclear magnetic resonance (MAS NMR) spectroscopy. ^7^Li MAS NMR spectra of both samples with different spinning speeds (0–20 kHz) are shown in Figure 1d. Peak width of NMR spectra is broader for rocksalt Li_2_TiS_3_, which is indicative of a diverse local environment for Li ions associated with cation disordering. These findings are consistent with each other; nanosized and disordered phases are formed by mechanical milling, and highly crystalline phase with a higher local symmetry is formed by subsequent heating process.

**Figure 1:**
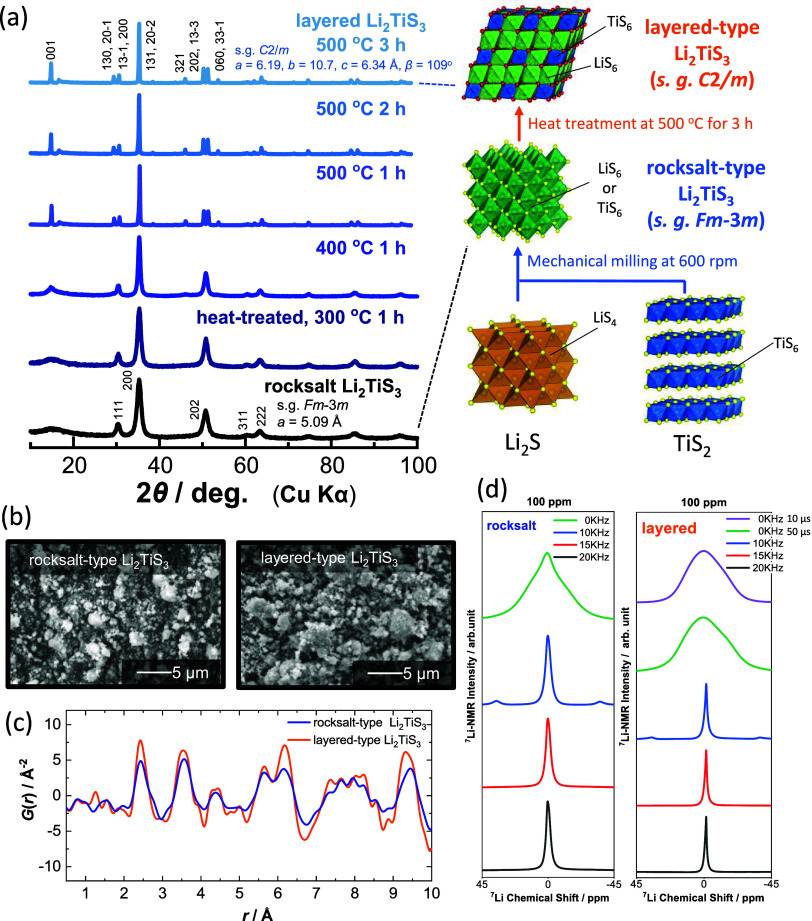
Synthesis and structural characterization of Li_2_TiS_3_ with ordered and disordered structures: (a) structural evolution of cation-ordered layered Li_2_TiS_3_ from cation-disordered rocksalt Li_2_TiS_3_ on heating, and a scheme of material synthesis is also shown. Comparison of (b) SEM images, (c) pair distribution functions, and (d) ^7^Li NMR spectra of ordered (layered) and disordered (rocksalt) Li_2_TiS_3_.

### Electrochemistry of Li_2_TiS_3_ and LiMn_0.5_Ti_0.5_S_2_ Polymorphs

Electrochemical properties of rocksalt and layered Li_2_TiS_3_ are compared, and galvanostatic charge/discharge curves of both samples are shown in Figure 2a. Layered Li_2_TiS_3_ is electrochemically inactive, whereas rocksalt Li_2_TiS_3_ shows superior electrode reversibility. Note that the layered sample shows a very small reversible capacity even after being charged to 4.5 V in Li cells. In contrast, layered LiMn_0.5_Ti_0.5_S_2_, which has been synthesized with the same methodology with layered Li_2_TiS_3_, is electrochemically active and shows a reversible capacity of 125 mA h g^–1^, corresponding to approximately 60% of the theoretical capacity based on Li contents in the host structure and one electron redox (LiMn_0.5_Ti_0.5_S_2_ → □Mn_0.5_Ti_0.5_S_2_ + Li^+^ + e^–^, herein “□” denotes a vacant octahedral site). Moreover, similar to Li_2_TiS_3_, disordered LiMn_0.5_Ti_0.5_S_2_ shows a better electrode performance compared with that of the layered sulfide. This difference originates from superior electronic conductivity for the rocksalt structure, as shown in Supporting Figure S2. A methodology of conductivity measurements for powder samples is found in the literature.^26^ Nearly 10 times higher electronic conductivity is observed for Li_2_TiS_3_ with the rocksalt structure. The resistivity of the samples was calculated to be 160 Ω·m for rocksalt Li_2_TiS_3_ and 1600 Ω·m for the layered Li_2_TiS_3_. It should be noted that the resistivity was measured using compressed powder pellets (area: 0.79 cm^2^, thickness: 1 mm) without sintering. The resistivity values obtained from compressed powder samples are expected to be higher than those of sintered samples due to grain boundary resistance and the presence of pores. The enrichment of structural defects with a higher concentration of grain boundary induced by high-energy mechanical milling may increase electronic conductivity. A similar phenomenon, the improvement of electronic conductivity for nanosized disordered oxides synthesized by mechanical milling, has also been reported.^26^ Electron delocalization, coupled with band broadening, is also expected because more complicated local structures are formed for materials with the cation-disordered rocksalt structure,^14,16^ as evidenced by NMR study (Figure 1d). In addition, ionic conductivity is influenced by the local structures of grain boundaries,^27^ and therefore, the structural defects affect ionic conductivity for these nanosized sulfides. Activation of anionic redox for Li_2_TiS_3_ with ordered and disordered structures is further discussed in the theoretical section.

**Figure 2:**
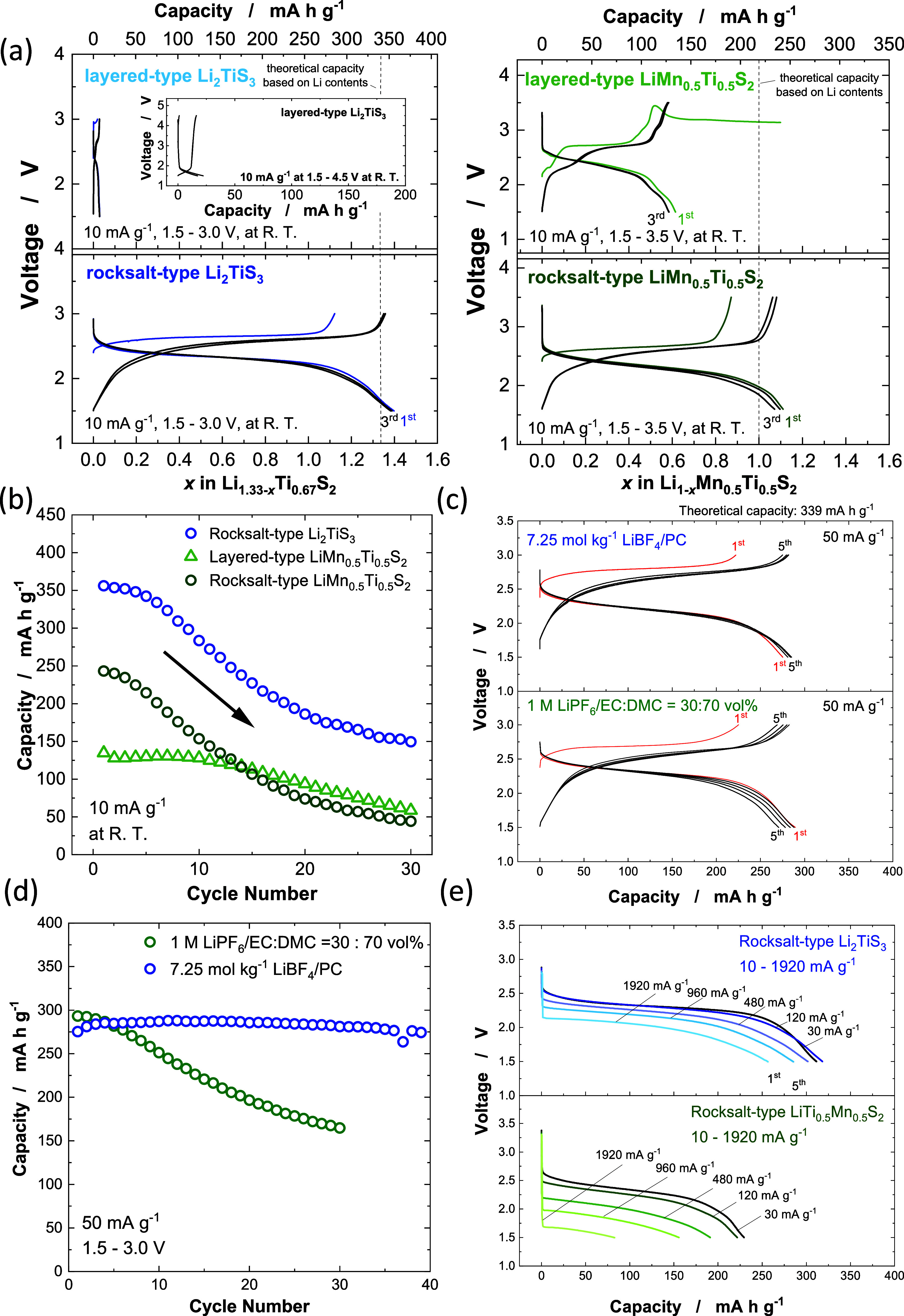
Electrochemical properties of Li-excess and stoichiometric Li sulfides: (a) Galvanostatic charge/discharge curves and (b) capacity retention of ordered (layered) and disordered (rocksalt) Li_2_TiS_3_ and LiMn_0.5_Ti_0.5_S_2_. Galvanostatic charge/discharge curves and capacity retention with different electrolyte solutions are also shown in (c) and (d), respectively. (e) Comparison of rate capability of disordered (rocksalt) Li_2_TiS_3_ and LiMn_0.5_Ti_0.5_S_2_ with a mass loading of 4–6 mg cm^–2^.

Initial charge capacities for both rocksalt sulfides are slightly smaller compared with the second cycle, which originates from partial oxidation of the samples. Similar phenomena are known for the samples with high surface area synthesized by high-energy mechanical milling.^26,28^ Such oxidation reaction during mechanical milling is mediated for the samples with lower electrode potential, <3 V vs. Li/Li^+^, presumably associated with oxidation by water and other protonated solvents coupled with hydrogen molecule generation. Although a large reversible capacity of 350 mA h g^–1^ (Figure 2a), which nearly corresponds to the theoretical capacity of Li_2_TiS_3_, is obtained for the sample with the rocksalt structure, its reversibility is rapidly lost on electrochemical cycles (Figure 2b). The capacity fading originates from the dissolution of sulfide ions into electrolyte solutions with polar solvents, and therefore, its reversibility of lithium-containing metal sulfides as electrode materials is significantly improved by using a polymer-based solid electrolyte.^15^ Similarly, the use of highly concentrated electrolyte solutions is also known to be beneficial to mitigate the dissolution of soluble species, like V and Mn ions because of the absence of free solvent molecules.^28,29^ Therefore, 7.25 mol kg^–1^ LiBF_4_ dissolved in propylene carbonate (PC)^30^ was used as an electrolyte solution. Although 7.25 mol kg^–1^ LiBF_4_/PC shows lower ionic conductivity (0.141 mS cm^–1^) because of high viscosity,^30^ much improved reversibility as electrode materials is obtained for the rocksalt Li_2_TiS_3_ cycled with the concentrated electrolyte (Figure 2c). Good capacity retention, no capacity fading for continuous 40 cycles, is achieved, as shown in Figure 2d.

It is anticipated that Li_2_TiS_3_ (Li_1.33_Ti_0.67_S_2_) shows inferior electronic conductivity compared with LiMn_0.5_Ti_0.5_S_2_ because Li_2_S is an insulator and the enrichment of Li ions in the host structure results in lowered electronic conductivity. Nevertheless, much better rate capability as electrode materials is observed for Li_2_TiS_3_ with the rocksalt structure (Figure 2e). Li_2_TiS_3_ delivers a discharge capacity of >250 mA h g^–1^ even at a rate of 1920 mA g^–1^. In contrast, a discharge capacity of <100 mA h g^–1^ at the same rate is observed for LiMn_0.5_Ti_0.5_S_2_. This fact indicates that discharge rate capability for both materials is limited by the percolative Li-ion conduction,^31^ and not by the electronic conduction. The enrichment of Li ions in the host structure results in improved percolative conduction of Li ions, leading to superior ionic conductivity and rate capability as electrode materials.

### Reaction Mechanisms of Li_2_TiS_3_ and LiMn_0.5_Ti_0.5_S_2_ with the Rocksalt Structure

To study the reaction mechanism of two different sulfides with the rocksalt structure, Li_2_TiS_3_ and LiMn_0.5_Ti_0.5_S_2_, an XRD study was used for electrochemically charged/discharged samples. As shown in Figure 3a, for Li_2–*x*_TiS_3_, a clear reduction in crystallinity is noted after charge to 3.0 V and full extraction of Li ions (also see Supporting Figure S3), suggesting that an amorphous phase is partially formed after oxidation in a Li cell. A similar observation was also reported in the literature.^11^ In contrast, higher crystallinity is retained for Li_1–*x*_Mn_0.5_Ti_0.5_S_2_, and the formation of the amorphous phase is relatively mitigated for the non-Li-excess and stoichiometric metal sulfide. However, both materials show higher crystallinity after full discharge (rereduction) to 1.5 V in a Li cell, suggesting that the amorphous phase formed on charge is recrystallized on discharge as a reversible process.

**Figure 3:**
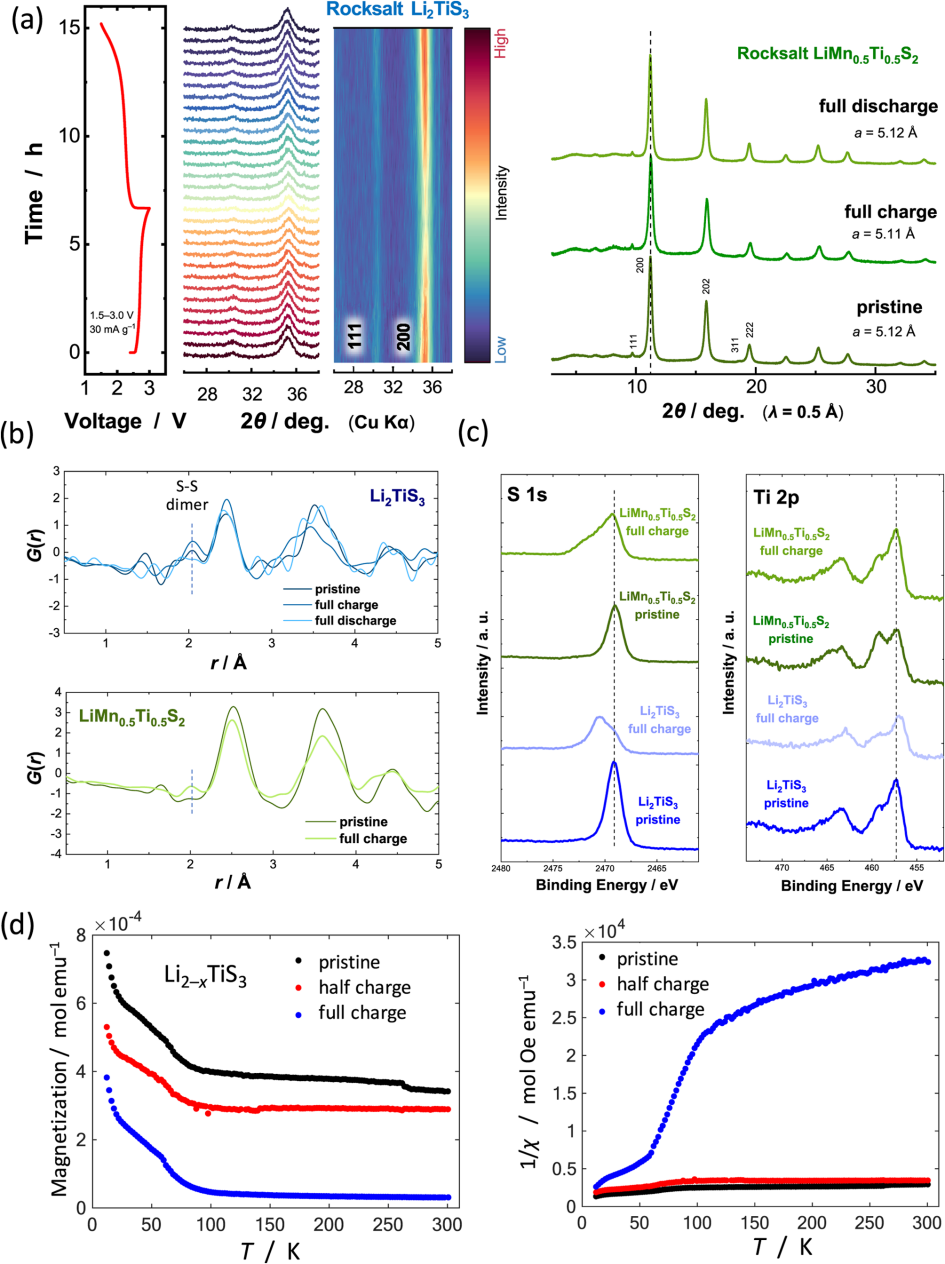
Reaction mechanisms of disordered (rocksalt) Li_2–*x*_TiS_3_ and Li_1–*x*_Mn_0.5_Ti_0.5_S_2_: (a) *In situ* XRD data of Li_2_TiS_3_ and *ex situ* synchrotron XRD data of LiMn_0.5_Ti_0.5_S_2_ during charge/discharge. Comparison of (b) pair distribution functions and (c) HAXPES spectra for both samples. (d) Results of magnetic measurements for disordered (rocksalt) Li_2–*x*_TiS_3_.

A local structure study by PDF analysis further reveals that the amorphous phase formation observed on charge clearly correlates with a S–S dimer formation, as shown in Figure 3b. The largest peak at 2.5 Å can be assigned to a chemical bond between Ti and S in Li_2_TiS_3_. The peak intensity at 2.05 Å is intensified after charge, which can be assigned as the formation of S–S dimers.^11^ This peak is also observed for the pristine sample of Li_2_TiS_3_ because partial oxidation during material synthesis is unavoidable as mentioned above (Figure 2a). However, after the discharge process to 1.5 V, the peak originating from the S–S dimers is clearly less intensified. Note that a similar peak at 2.05 Å also appears for Li_1–*x*_Mn_0.5_Ti_0.5_S_2_ after charge to 3.0 V, but the more intensified peak is observed for Li_2–*x*_TiS_3_, which is consistent with the fact that the non-negligible fraction of the amorphous phase is formed for Li_2–*x*_TiS_3_ compared with Li_1–*x*_Mn_0.5_Ti_0.5_S_2_ (Figure 3a).

Charge compensation by the solid-state redox reaction of sulfide ions was further examined by hard X-ray photoelectron spectroscopy (HAXPES). Compared with soft X-ray PES, information from relatively thicker regions (∼10 nm from the surface) can be obtained by HAXPES because of the higher kinetic energy for ejected electrons (photoelectrons). For S 1s HAXPES spectra of the pristine samples, a clear peak centered at 2469 eV is observed for both samples (Figure 3c), which can be assigned as a sulfide ion, S^2–^. After charge, a new peak appears at a higher binding energy region, ∼2471 eV, and the intensity of the original peak for sulfide ions at 2469 eV is clearly weakened associated with oxidation of sulfide ions followed by partial dimerization of sulfur species. The original peak of sulfide ions is clearly enriched for Li_1–*x*_Mn_0.5_Ti_0.5_S_2_ after charge to 3.0 V, which is consistent with the results of XRD and PDF analysis, *i.e*., the partial mitigation of S–S dimerization for Li_1–*x*_Mn_0.5_Ti_0.5_S_2_. In contrast to S 1s HAXPES spectra, no clear change is found in Ti 2p HAXPES spectra (Figure 3c), indicating that Ti ions are not responsible for the charge compensation as expected from the nature of Ti^4+^ ions with d^0^ configuration.

To identify redox species in Li_2–*x*_TiS_3_, the magnetic susceptibility (*χ*) is measured before and after charge.^32^ For Na_2–*x*_Mn_2_O_7_, the hole formation and stabilization in anionic species were efficiently detected by the magnetic measurement.^33^ A similar methodology can be applied for Li_2–*x*_TiS_3_ as shown in Figure 3d. For the fully charged sample to 3.0 V, a slope of 1/*χ* as a function of temperature below 50 K and above 100 K is clearly large compared with the pristine and half-charged samples, indicating that a value of the Curie constant is the smallest among the three samples. When the holes are electrochemically formed and stabilized in sulfide ions, an increase in values of Curie constant is anticipated because of the absence of magnetic active species in pristine sample.^33^ This finding indicates that holes cannot be stabilized in Li_2–*x*_TiS_3_, and S–S dimerization is the dominant process upon charge in Li cells. A nonlinear change in 1/*χ* between 50 and 100 K suggests a phase transition at this temperature range, which is out of scope for this article.

### X-ray Absorption Study on Disordered Li_2–*x*_TiS_3_ and Li_1–*x*_Mn_0.5_Ti_0.5_S_2_

To further elucidate the charge compensation process by the solid-state redox reaction of sulfide ions, X-ray absorption (XAS) spectroscopy was adopted. S K-edge and Ti K-edge XAS spectra collected with a fluorescence yield mode for Li_2–*x*_TiS_3_ and Li_1–*x*_Mn_0.5_Ti_0.5_S_2_ are shown in Figure 4a. Two major peaks, a pre-edge peak centered at 2470 eV and a broad peak at 2477 eV, are observed, and a similar XAS profile is observed for Li_2_S.^34^ A clear peak shift to a higher energy region is observed for pre-edge spectra at the S K-edge of Li_2–*x*_TiS_3_ on charge, which is indicative of sulfur oxidation, and this result is consistent with the observation of HAXPES (Figure 3c). Peak top energy is shifted by 1 eV coupled to peak sharpening after full charging for Li_2–*x*_TiS_3_. Moreover, no change is observed for Ti K-edge XAS spectra after discharge, indicating that Ti ions cannot be electrochemically reduced on discharge. These observations conclude that only sulfide ions are responsible for charge compensation without the contribution of Ti ions as redox species. For Li_1–*x*_Mn_0.5_Ti_0.5_S_2_, a similar observation is noted for S K-edge spectra. Moreover, the oxidation of Mn^2+^ ions is not evidenced after the charge to 3.0 V in Li cells. Similarly, for Li_1–*x*_Fe_0.5_Ti_0.5_S_2_, the oxidation of Fe^2+^ ions is not evidenced as reported in the literature.^15^ When the peak profiles of fully charged samples for Li_2–*x*_TiS_3_ and Li_1–*x*_Mn_0.5_Ti_0.5_S_2_ are compared, a larger pre-edge peak at 2471 eV is observed for Li_2–*x*_TiS_3_, which is consistent with the experimentally observed larger charge capacity and thus more pronounced oxidation of S species in Li_2–*x*_TiS_3_ (Figure 2). These results suggest that sulfide ions are mainly responsible for charge compensation without the contribution of transition metal ions with d electrons. This finding is also supported by the fact that similar operation voltage, ∼2.5 V, is observed for both Li_2–*x*_TiS_3_ and Li_1–*x*_Mn_0.5_Ti_0.5_S_2_ (Li_1–*x*_Fe_0.5_Ti_0.5_S_2_ ^15^) as shown in Figure 2a, regardless of the presence of other transition metal ions with d electrons. The d electron bands for Mn and Fe ions for sulfide-based materials are found at a lower energy region compared with sulfur 3p bands, leading to a unique nature as electrode materials, which cannot be observed for conventional layered oxides with transition metal ions such as LiCoO_2_ and LiNiO_2_. Therefore, band broadening for sulfide-based materials with the disordered structure, presumably coupled with the formation of defect bands near Fermi level for nanosized crystalline samples, leads to improved electronic conductivity.

**Figure 4:**
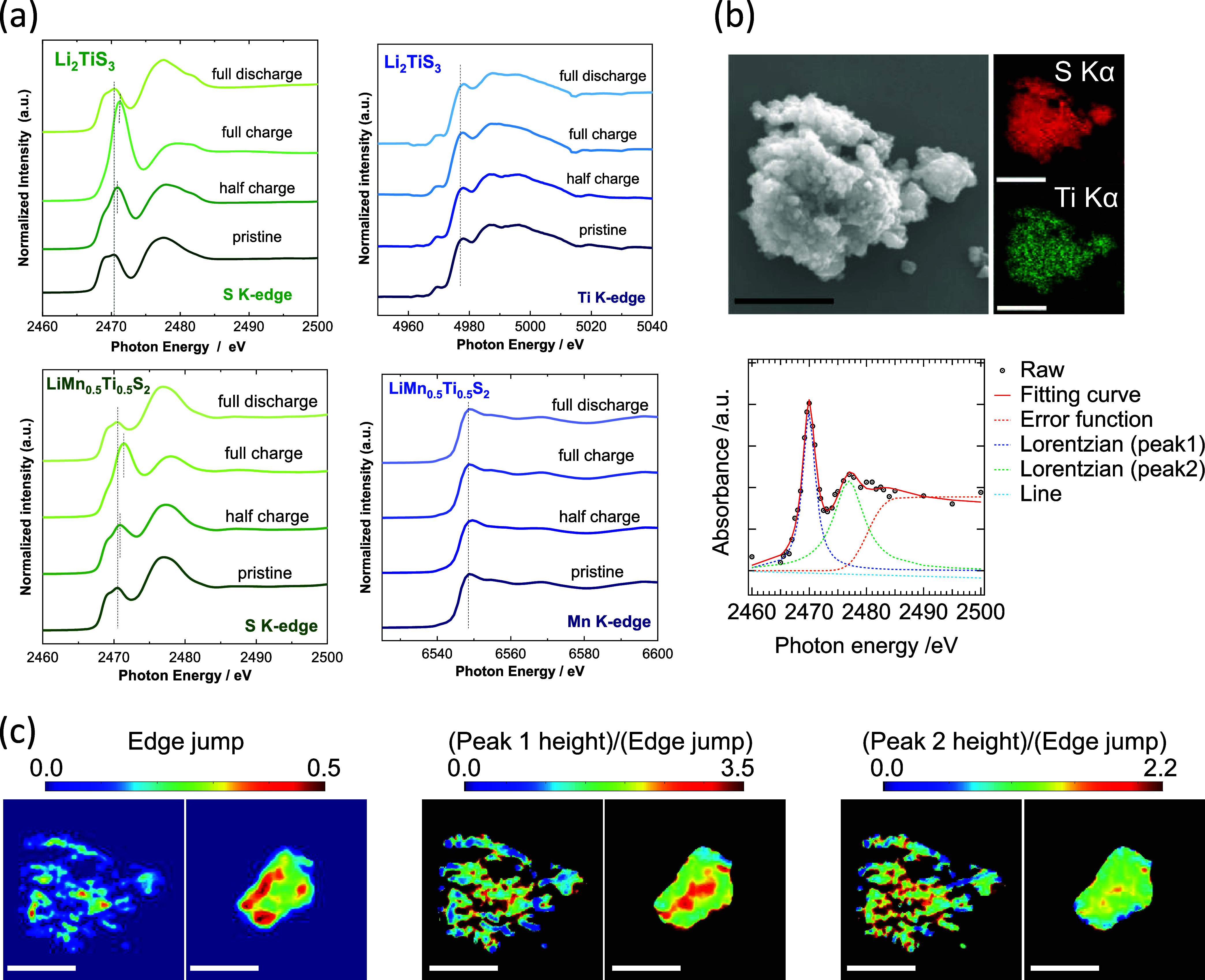
Study of changes in electronic structures by XAS spectroscopy: (a) Comparison of XAS spectra for disordered (rocksalt) Li_2–*x*_TiS_3_ and Li_1–*x*_Mn_0.5_Ti_0.5_S_2_, (b) SEM/EDX spectra of half-charged Li_2–*x*_TiS_3_ with the disordered structure and an example of curve fitting for a reconstructed absorption spectrum of full-charged Li_2–*x*_TiS_3_, (c) chemical state maps of edge-jumps and normalized peak heights of (left) half-charged and (right) fully charged samples obtained by X-ray spectroscopic ptychography measurements. Scale bars correspond to 2.5 μm.

To further validate the electronic and chemical natures of Li_2–*x*_TiS_3_ with a high spatial resolution (∼100 nm), X-ray spectroscopic ptychography measurements were conducted at the S K-edge. Half-charged and fully charged samples were obtained by chemical oxidation. To confirm distributions of S and Ti ions in the oxidized particles, prior to the ptychography study, SEM/EDX spectroscopy was applied (Figure 4b and S4a), and the results indicate that S and Ti ions are uniformly found in the particles without segregation. The detailed methodology for ptychography analysis is described in our publication.^35,36^ Reconstructed absorption and reconstructed phase images are shown in Supporting Figure S4b with 20 nm pixel size resolution. The particle shapes in the phase images, which contain information on electron amounts, for both samples are similar to those of SEM images. From the reconstructed absorption and phase images, spatially resolved S K-edge XAS and phase spectra are also obtained in Supporting Figure S4c. The spatially resolved XAS spectra show similar profiles to those of the conventional spectra (Figure 4a). Chemical state mapping was obtained by the curve-fitting analysis of spatially resolved XAS spectra and the phase images. An example of a curve-fitting analysis is shown in Figure 4b. Two major peaks, peaks 1 and 2, were fitted, and chemical state mapping was obtained by the normalization of peak intensities by edge jump, as shown in Figures 4c and S5. The average energy of peak 2 position is increased for the fully charged sample compared with the half-charged sample (Supporting Figure S5), indicating that average oxidation states of sulfide ions are increased for the fully charged sample. Moreover, nonuniformity for the chemical oxidation states for sulfur species is noted for both samples, suggesting that different local structures for sulfide ions are formed for the charged samples. Such nonuniformity of electrochemical reactions for the compounds with the disordered structure is further discussed in the theoretical section.

### Theoretical Analysis of Delithiation from Li_2_TiS_3_ with Ordered and Disordered Structures

For theoretical study, two types of structures, ordered layered and disordered rocksalt structures, are considered for Li_2_TiS_3_. The Li/Ti configuration for layered Li_2_TiS_3_ structure was referred to that of layered Li_2_TiO_3_.^37^ On the other hand, special quasi-random structure (SQS) approach^38^ was performed to mimic random configuration for Li_2_TiS_3_ cell consisting of a relatively small number of atoms. In this study, only two-body interactions are considered between adjacent cation–cation pairs (Li–Li, Li–Ti, and Ti–Ti), and candidate structures are generated by the Metropolis Monte Carlo simulation. Figure 5a displays the obtained SQS-derived cell, Li_24_Ti_12_S_36_, and distribution of coordination numbers (CNs) of the nearest neighbor Li–Li interactions as an example (Figure 5b). A good accordance of the CN distribution is indicated between SQS structure and ideal random structure. The energy of ordered Li_2_TiS_3_ is 0.334 eV per Li_2_TiS_3_ unit lower (more stable) than that of disordered Li_2_TiS_3_, which corresponds to the order–disorder phase transition temperature at approximately 2030 K by solely considering the configurational entropy effect arising from Li/Ti arrangements. According to Lun et al.,^39^ the temperature of 2573 K corresponds to the high-energy conditions generated with ball milling in Li–Mn–O–F chemical species. This result is also consistent with the fact that the disordered phase synthesized by high-energy milling is a metastable phase and changes into the ordered phase by heating (Figure 1a). However, the difference between the estimated phase transition temperature derived from the configurational entropy and the actual ball-milling synthesis temperature is relatively small. As a result, uniform formation of the disordered phase may not be fully achieved during the synthesis, potentially leading to a certain degree of compositional inhomogeneity. The experimentally observed nonuniform features at the particle scale in the ptychography results are thus likely to reflect the inhomogeneous distribution of the disordered phase characterized by SLi_6_ coordination environments. Because the electrochemical Li extraction is performed around room temperature, the SQS approach that assumes high-temperature extreme configuration is not appropriate to determine Li/vacancy arrangement of delithiated Li_2–*x*_TiS_3_ phase. Instead, the genetic algorithm (GA) approach is used to optimize the Li/vacancy configuration in the fixed ordered and disordered □_2_TiS_3_ flameworks (□ denotes vacant sites). The superstructures of Li_32(1–*x*)_Ti_16_S_48_ and Li_24(1–*x*)_Ti_12_S_36_ are used for ordered and disordered flameworks,^40–43^ respectively. Details on the GA optimization are described elsewhere.^40–43^

**Figure 5:**
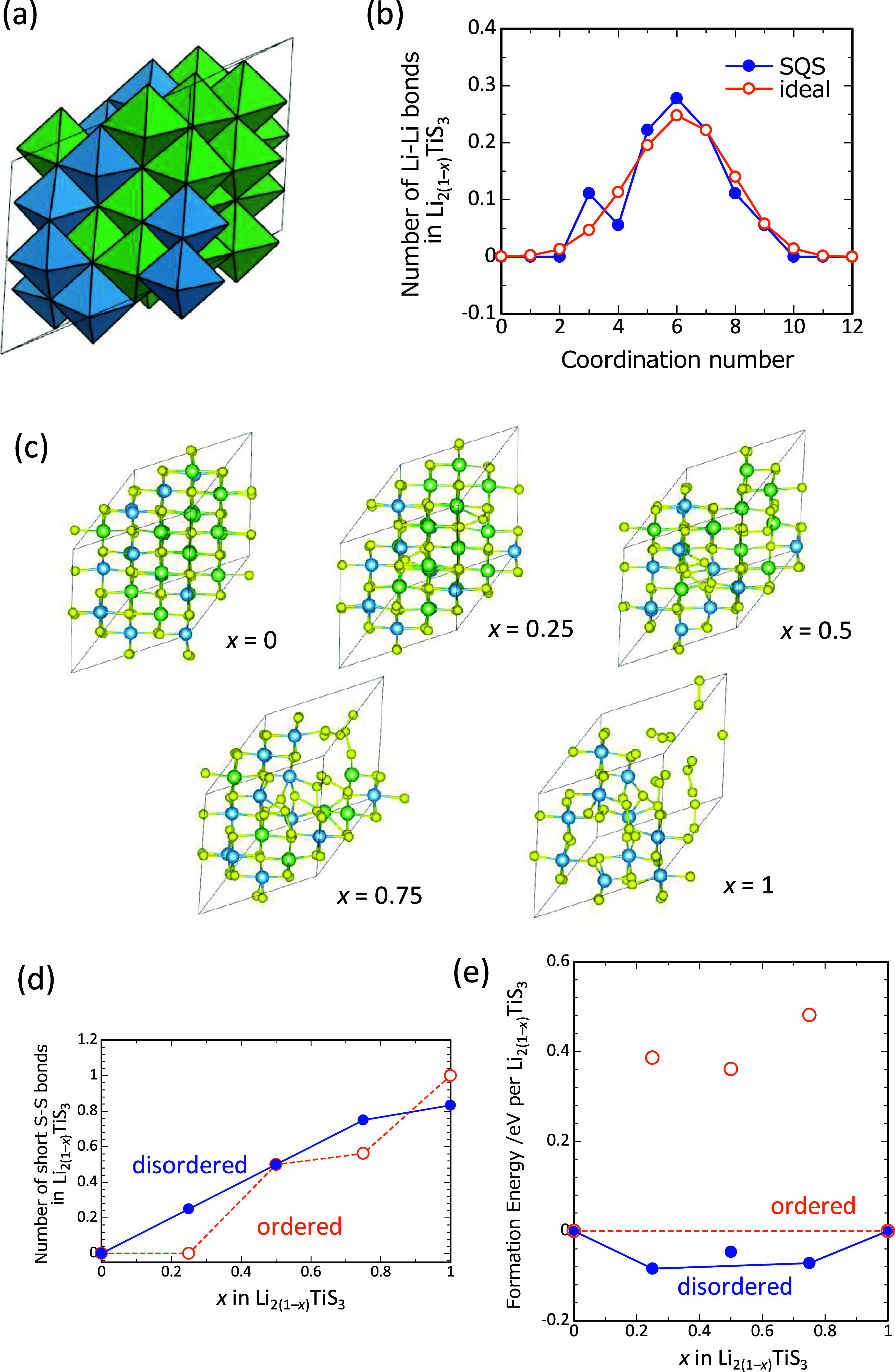
(a) Crystal structure of disordered rocksalt Li_2_TiS_3_ derived from special quasi-random structure (SQS) approach. The simulation cell composition is Li_24_Ti_12_S_36_. (b) Coordination number distribution between Li and nearest neighbor Li ions (Li–Li bonds). Blue and orange lines correspond to SQS-derived simulation cell (panel (a)) and ideal random structure which considers random Li/Ti arrangement using large cell consisting of 1 000 000 cation sites. Coordination number distribution for Li–Ti and Ti–Ti interactions are also considered for simulation cell building derived from SQS approach. (c) Evolution of crystal structure with delithiation process from disordered sample cell, i.e., Li_24(1–*x*)_Ti_12_S_36_ (*x* = 0, 0.25, 0.5, 0.75, and 1.0), where Li/vacancy arrangement for intermediate compositions in the structure inputs are derived from genetic algorithm (GA) approach. Yellow, light-blue, and green spheres indicate sulfur, titanium, and lithium atoms, respectively. (d) Number of short S–S bonds (bond length of S–S is less than 2.2 Å) as a function of composition *x* in Li_2(1–*x*)_TiS_3_ (*x* = 0, 0.25, 0.5, 0.75, and 1.0). Blue and orange lines represent delithiation process from disordered and ordered structures, respectively. (e) DFT calculated formation energies as a function of composition *x* in Li_2(1–*x*)_TiS_3_. Blue and orange symbols represent DRS and LRS structures, respectively. Tie lines correspond to convex hull.

Figure 5c shows GA optimized disordered Li_2(1–*x*)_TiS_3_ (*x* = 0, 0.25, 0.5, 0.75, and 1), and Figure 5d summarizes variation of the number of S–S bonds whose length is less than 2.1 Å as a function of composition *x* for both ordered and disordered structures. As the delithiation proceeds, the number of short S–S bond formations, i.e., formation of disulfide (S_2_^2–^), increases. Hence, the disulfide formation by the oxidation reaction of sulfide ions, such as 2S^2–^ → S_2_^2–^ + 2 e^–^, is mediated during electrochemical Li removal. Note that trisulfide (S_3_^2–^) and tetrasulfide (S_4_^2–^) ions are also found, as shown in Figure 5c. Short S–S bonds are not formed for the composition *x* = 0.25 with ordered structure, indicating another oxidation reaction mechanism as discussed later.

Phase stability of partially delithiated compositions was further evaluated by plotting formation energies, Δ*E*_f_, as displayed in Figure 5e. The formation energy is computed using total electron energies of the end compositions (*x* = 0 and 1) as references.

1 $$\Delta E_{f}\;\left({\mathrm{Li}}_{2\left(1-x\right)}\;{\mathrm{TiS}}_{3}\right)=E_{0}\;\left({\mathrm{Li}}_{2\left(1-x\right)}\;{\mathrm{TiS}}_{3}\right)-\left(1-x\right)\;E_{0}\;\left({\mathrm{Li}}_{2}{\mathrm{TiS}}_{3}\right)-x\;E_{0}\;\left({\mathrm{TiS}}_{3}\right)$$

where *E*_0_ (*X*) corresponds to the total electron energy of composition *X*. Formation energies of intermediate compositions in ordered Li_2(1–*x*)_TiS_3_ are positive, indicating two-phase coexistence reaction between fully lithiated (*x* = 0) and delithiated (*x* = 1) compositions. On the other hand, formation energies are slightly negative for the partially delithiated phase of disordered Li_2(1–*x*)_TiS_3_, suggesting that the reaction is likely to proceed in a solid-solution manner. This result is also consistent with the finding by an experimental study in Figure 3a. The calculated voltage profiles are shown in Supporting Figure S6. The onset voltage of delithiation for both ordered and disordered Li_2_TiS_3_ is calculated to be ∼2.2 V. Gradual increase of voltage up to ∼2.5 V is visible for disordered Li_2_TiS_3_ because of solid-solution reaction, while the voltage for ordered Li_2_TiS_3_ is constant due to the two-phase coexistence reaction. The calculated voltage for the delithiation from ordered Li_2_TiS_3_ via metastable solid solution route is ∼3 V, which is more than ∼0.8 V higher than that of the equilibrium two-phase coexistence reaction. However, the fact is that ordered Li_2_TiS_3_ is electrochemically inactive, ∼4.5 V in Li cells (Figure 2a), suggesting that anionic redox of the ordered phase is hindered by a kinetic limitation as discussed in the next section.

### Nonuniform Li Extraction for Li_2–x_TiS_3_ with the Cation-Disordered Structure

Bader charge analysis was performed for disordered and ordered Li_2–*x*_TiS_3_ to elucidate oxidation reaction mechanisms including metastable delithiated phases (Figure 6a,b). In both phases, Bader charges of Ti ions are almost constant regardless of composition *x*, indicating that no electron exchange reaction occurs at Ti ions, keeping the Ti^4+^ (d^0^) electronic configuration during delithiation. On the other hand, a gradual increase in averaged Bader charges of sulfide ions is observed, indicating oxidation of sulfur species during delithiation. In detail, Bader charges of S ions are almost the same as ca. −1.2 at composition *x* = 0 (before delithiation), suggesting little local structure configuration effect in the disordered phase. Bader charges for several S atoms in disordered □_0.5_Li_1.5_TiS_3_ (*x* = 0.25) show discontinuous increases ranging from −1.0 to 0, which is attributed to the formation of disulfide ions (S_2_^2–^) and trisulfide ions (S_3_^2–^) as shown in Figure 6c. On the other hand, Bader charges for all the S ions show a slight increase in metastable ordered □_0.5_Li_1.5_TiS_3_ (*x* = 0.25) as shown in Figure 6b, which corresponds to the no short S–S bond formation. In both disordered and ordered structures, an electronic exchange for the oxidation reaction proceeds by the formation of disulfide, trisulfide, and tetrasulfide ions at composition *x* ≥ 0.5.

**Figure 6:**
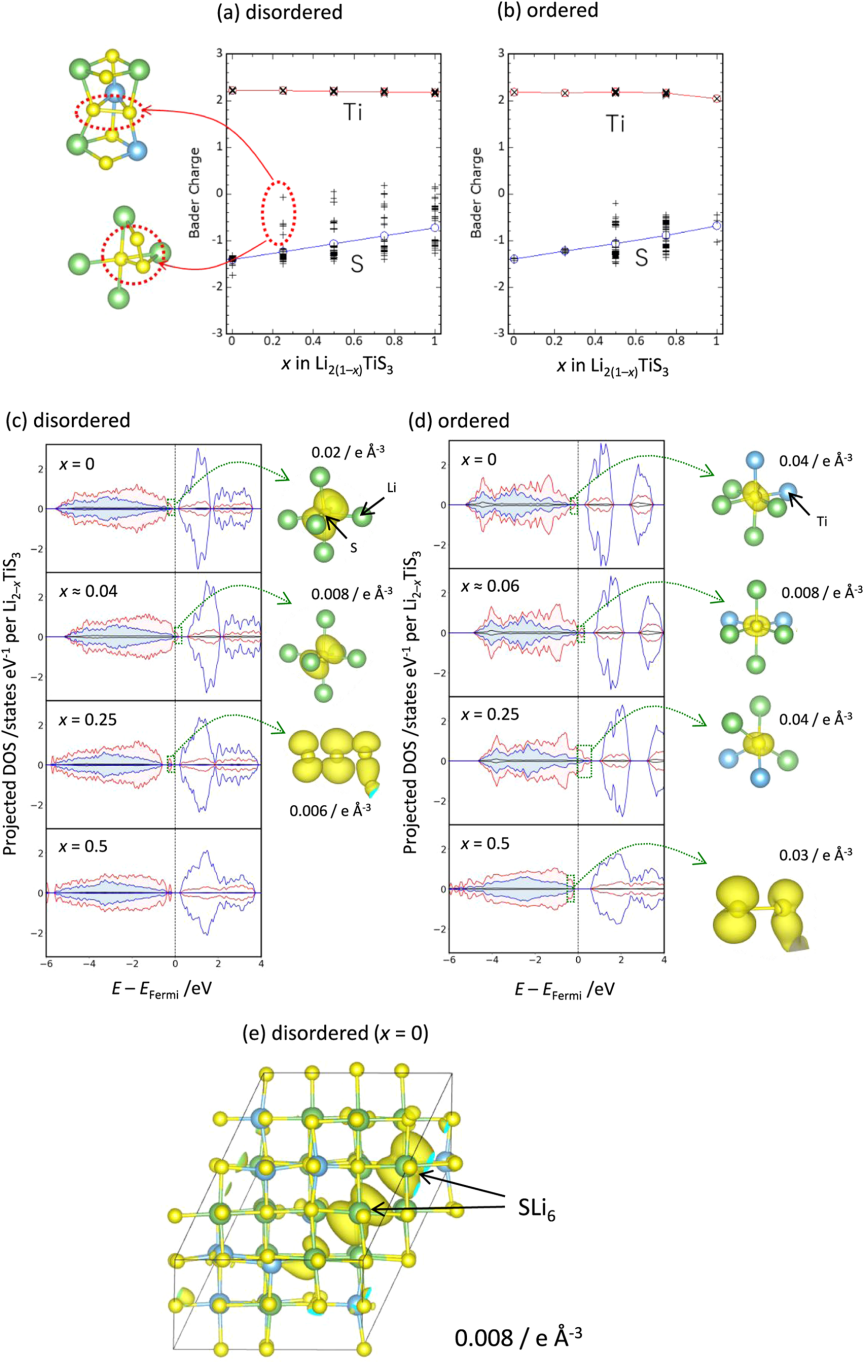
(a) Bader charges for Ti and S ions as a function of composition *x* in (a) disordered and (b) ordered Li_2(1–*x*)_TiS_3_. Cross symbols refer to an individual Ti or S ion, while lines correspond to averaged Bader charges for Ti or S ions. Projected density of states (PDOS) for Ti and S in (c) disordered and (d) ordered Li_2(1–*x*)_TiS_3_ (*x* = 0, 0.25, and 0.5). Blue and red lines correspond to PDOS for Ti and S, respectively. (c–e) Partial electron/hole densities around Fermi level (marked as hatched line) are visualized as isosurface projections at the side of PDOS diagrams at different isovalues. Yellow, light-blue, and green spheres correspond to S, Ti, and Li atoms, respectively.

Projected density of states for Ti and S atoms and visualized electron (hole) densities near the Fermi level for disordered and ordered Li_2(1–*x*)_TiS_3_ (*x* = 0, 0.25, and 0.5) are also shown in Figure 6c,d. In both structures, valence and conduction bands are composed of mainly S 3p and partially Ti 3d orbitals, respectively. Bandgaps for disordered and ordered Li_2_TiS_3_ are 0.65 and 0.79 eV, respectively, by evaluating the difference in the total energies between the system containing electron, hole, and neutral cell.^44,45^ Such band gap broadening is expected to originate from diverse local structures for disordered Li_2_TiS_3_. A similar discussion was provided for ordered and disordered Li_3_NbS_4_ ^14^ and vanadium-based oxides.^16^ Furthermore, the presence of a unique local environment for sulfide ions in the disordered phase, which cannot be formed for ordered Li_2_TiS_3_, is noted. The valence band maximum (VBM), where electron extraction occurs by electrochemical delithiation, is visualized as partial electron densities for both disordered and ordered Li_2_TiS_3_ at composition *x* = 0. The visualized electron density corresponding to VBM of disordered Li_2_TiS_3_ is localized for the specific two sulfide ions as shown in Figure 6e (also see Figure 6c, *x* = 0), and both sulfide ions are surrounded by 6 Li ions. This is due to a nonbonding nature between Li and sulfide ions, while partially covalent bonding formation between Ti and sulfide ions reduces the energy level of the occupied orbital states. This unique local environment for sulfide ions, SLi_6_, is formed only for the disordered phase. The electrons (or holes) in the orbitals near the VBM appearing in the SLi_6_ environment seem to be localized based on the figure. Therefore, the orbital density at each energy level projected onto the radius of the augmentation sphere used in the projector-augmented wave (PAW) method was evaluated as a measure of localization (Supporting Figure S7a,b). A localization value close to 1 indicates strong localization, whereas a smaller value suggests delocalization. The results showed that the localization value near the valence band (S 3p orbital) is approximately 0.6 for both disordered and ordered Li_2_TiS_3_, with no significant difference observed. In contrast, for the corresponding oxide model of disorder Li_2_TiO_3_ based on the disordered Li_2_TiS_3_, crystal structure, the localization value increased to 0.8 (Supporting Figure S7c). Considering that the PAW sphere radius for oxygen is smaller than that for sulfur, the electrons in the oxide are considered to be more localized around the O atoms at the OLi_6_ configuration. In addition, upon slight Li extraction from Li_2_TiS_3_ (in Figure 6c, disordered, and in Figure 6d, ordered models), the compositions correspond to approximately *x* ∼ 0.04 and *x* = 0.06, respectively (hereafter, such slightly delithiated compounds are referred to as Li_2−δ_TiS_3_), the PDOS reveals that hole states appear near the Fermi level (*E* – *E*_F_ < 0.3 eV) in both disordered and ordered models. These hole states match the orbitals near the VBM prior to delithiation and originate from the S 3p orbitals (Figures 6c,d and S8). When delithiation starts in disordered Li_2_TiS_3_ by electrochemical oxidation, Li ions are nonuniformly extracted from SLi_6_ environment, and such nonuniformity for Li extraction is also consistent with the experimental finding by ptychography study (Figure 4c). On the other hand, all the sulfide ions are surrounded by 4 Li and 2 Ti ions in ordered Li_2_TiS_3_. Partial electron density for VBM of ordered Li_2_TiS_3_ (*x* = 0) shown in Figure 6d indicates an S 3p-derived orbital. As reported, 180° Li–O–Li configuration is the primary reason for electrochemically active O 2p orbital formation due to nonbonding interaction, and this nonbonding (localized) O 2p orbital forms along the Li–O–Li direction.^46,47^ However, the visualized partial electron density around VBM does not show dumbbell- or ellipse-shaped nonbonding S 3p orbital along the Li–S–Li axis (Figure 6d). Therefore, unlike oxides, in sulfides, the S 3p orbitals do not appear to be localized even when Li–S–Li configurations are formed. This is also supported by the lower localization factor observed in sulfides compared to oxides (Supporting Figure S7). Furthermore, in the disordered Li_2−δ_TiO_3_ oxide, hole states formed upon slight delithiation appear approximately 0.4 eV above the Fermi level and are observed exclusively in the down-spin channel (Supporting Figure S8). This spin asymmetry suggests the presence of strong electron–electron Coulomb interactions within the partially occupied orbitals. Such spin-polarized localization of holes is characteristic of systems with strong electronic correlations, where intraorbital Coulomb repulsion leads to spin splitting and preferential occupancy of a single-spin channel. This behavior is commonly captured in correlated-electron models such as the Hubbard model and reflects the Mott-like nature of the hole states.^48,49^ Therefore, polaronic electron conduction is likely to proceed in disordered Li_2– *x*_TiO_3_ oxide electrode as reported in various oxide electrodes.^50–52^ In contrast, in the corresponding disordered sulfide Li_2−δ_TiS_3_, there was no significant difference in the DOS between spin-up and spin-down states, and the band structure was metallic-like (in fact, one of the bands was found to cross the Fermi level). These observations imply that electron correlation effects are weak and that electrical conductivity in the sulfide is governed by band conduction. Note that the absolute values of the spin moments, which are defined as the difference between spin-up and spin-down components within the augmentation spheres of each atom, were found to be less than 0.0001 μ_B_ throughout the composition range 0 ≤ *x* ≤ 0.5 in the DFT calculations performed for Li_2–*x*_TiS_3_. This result is consistent with the nearly identical shapes of the spin-up and spin-down PDOS shown in Figure 6c. Furthermore, the magnetic susceptibility measurements shown in Figure 3d exhibit paramagnetic behavior down to near absolute zero, in good agreement with the nonmagnetic nature suggested by the present DFT results. A similar trend was also observed in layered-type and ordered Li_2−δ_TiS_3_. However, the hole states tended to separate from the valence band. This indicates that initial delithiation in disordered Li_2_TiS_3_ results in the formation of metallic-like bands and relatively high electronic conductivity, whereas ordered Li_2_TiS_3_ exhibits a tendency to open a gap at the Fermi level, leading to lower electronic conductivity. Taken together, these findings are consistent with the electrochemical inactivity of ordered Li_2_TiS_3_ (Figure 2a) and the experimental results shown in Supporting Figure S2.

The difference in structures, disordered and ordered phases, and (non)­uniformity of delithiation for both phases also change dimerization processes for sulfide ions on oxidation. At the composition *x* = 0.25, a localized band (occupied) is formed in disordered □_0.5_Li_1.5_TiS_3_, and the visualized partial electron density indicates that the localized band is attributed to the trisulfide ion, S_3_^2–^ (Figure 6c, also see Figure 5c). Meanwhile, a metallic-like band is formed due to electron extraction (hole creation) from the S 3p band in ordered □_0.5_Li_1.5_TiS_3_ (*x* = 0.25) as follows the rigid-band model (Figure 6d). This theoretical finding indicates no formation of short S–S bonds for ordered □_0.5_Li_1.5_TiS_3_. The existence or absence of short S–S bonds in both phases is owed to vacancy configurations and structural distortions after delithiation. Li vacancies are selectively formed and enriched around the S ions coordinated with 6 Li ions in disordered □_0.5_Li_1.5_TiS_3_, and the enriched vacant sites allow to cause structural distortions to form S–S bonding. In the computational model of disordered □_0.5_Li_1.5_TiS_3_ (*x* = 0.25), the formation of S–S dimers opens a band gap, suggesting that the metallic-like band formation observed in disordered Li_2−δ_TiS_3_ may be lost in this configuration. However, not all charge transfer reactions are necessarily associated with dimer formation. It is plausible that regions supporting metallic-like band formation and electron delocalization persist via the formation of hole states that do not involve dimerization. Moreover, the formation of S–S dimers contributes to phase stability from a thermodynamic perspective, potentially facilitating smooth charge transfer reactions (Supporting Figure S7). Contrarily, isolated Li vacancies and holes are formed in ordered □_0.5_Li_1.5_TiS_3_, and thus local structural distortions are suppressed. Ordered Li_2_TiS_3_ is, therefore, electrochemically inactive, because of kinetic limitations. Note that S–S distances in ordered Li_2_TiS_3_ are ∼3.53 Å, which is much longer than that O–O distance (∼2.83 Å) in Li_2_MnO_3_. Hence, larger structural distortions are required to form S–S bonds in sulfide-based compounds. This finding is also consistent with the fact that the crystallinity is drastically lowered after Li extraction for disordered Li_2–*x*_TiS_3_ (Figures 3a and S3). Further Li removal and enrichment of vacant sites result in the structural distortion and formation of disulfide, trisulfide, and/or tetrasulfide ions for both phases, and thus the two PDOS are similar between disordered and ordered □LiTiS_3_ (*x* = 0.5).

## Conclusions

Li-excess and stoichiometric metal sulfide systems are targeted as high-capacity electrode materials with anionic redox, and the activation mechanisms of anionic redox for these sulfide systems are systematically studied with different characterization methodologies and theoretical approach. These findings derived by joint theoretical and experimental study conclude that structural disordering with different local structures, including the unique environment of SLi_6_ configuration formed only in the disordered structure, effectively activates anionic redox, which originates from high electronic conductivity associated with electron delocalization for sulfur 3p orbitals even without conductive d electrons coupled with easier structural distortion and S–S dimerization on delithiation. Although higher solubility to electrolyte solutions for the sulfide system is a practical problem as electrode materials, this problem is effectively mitigated by the use of highly concentrated electrolyte solutions, leading to a large reversible capacity, >300 mA h g^–1^, with highly reversible anionic redox. The insights potentially result in the future development of high-energy and cost-effective electrode materials with the reversible redox reaction of anionic species used in host structures.

## Supplementary Material



## Notes

XRD patterns of precursors, CV data for powdered samples of rocksalt and layered Li_2_TiS_3_, original *in situ* XRD data of Li_2–*x*_TiS_3_, SEM/EDX data of fully charged Li_2–*x*_TiS_3_ with the disordered structure, reconstructed phase and absorption images, phase, XAS spectra, chemical state maps, edge-jumps, and normalized peak position and heights, obtained by X-ray ptychography for half-charged and fully charged Li_2–*x*_TiS_3_ with the disordered structure, DFT calculated voltage profile as a function of composition *x* in Li_2–*x*_TiS_3_, electron localization factors and density of states for ordered/disordered Li_2_TiS_3_ and disordered Li_2_TiO_3_, magnified views of the density of states (DOS) near the Fermi level for disordered Li_2−δ_TiO_3_ and ordered/disordered Li_2−δ_TiS_3_ with isosurface projections of charge density (PDF)
