# Supplementary material for: Activation of Anionic Redox for Stoichiometric and Li-Excess Metal Sulfides through Structural Disordering: Joint Experimental and Theoretical Study
Source: J Am Chem Soc. 2025 Jul 15;147(30):26238–53. doi: 10.1021/jacs.5c04018 (PMC12314909; doi:10.1021/jacs.5c04018)
Supplement: Supplementary file 1 [file ja5c04018_si_001.pdf]

Supporting Information for

Activation of anionic redox for stoichiometric and Li-excess metal sulfides through structural disordering: joint experimental and theoretical study

Miyuki Shinoda,[1] Koki Matsunoshita,[2] Masanobu Nakayama,[2] Satoshi Hiroi,[3] Koji Ohara,[3, 4, 5] Masaki Abe,[4, 6, 7, 8] Nozomu Ishiguro,[4, 6] Yukio Takahashi,[4, 6, 8, 9] Gen Hasegawa,[5] Naoaki Kuwata,[5] Tsukasa Iwama,[5, 10] Takuya Masuda,[5, 10] Kosuke Suzuki,[11] Hirofumi Ishii, [12] Yu-Cheng Shao,[12] Daisuke Shibata,[13] Akinori Irizawa,[13] Toshiaki Ohta,[13] Itsuki Konuma,[1] Teppei Ohno,[1] Yosuke Ugata,[1, 14] and Naoaki Yabuuchi[1, 14]*

[1]Department of Chemistry and Life Science, Yokohama National University, 79-5 Tokiwadai, Hodogaya-ku, Yokohama, Kanagawa 240-8501, Japan

[2]Department of Advanced Ceramics, Nagoya Institute of Technology, Gokiso-cho, Showa-ku, Nagoya, Aichi 466-8555, Japan

[3]Faculty of Materials for Energy, Shimane University, 1060 Nishikawatsu-cho, Matsue, Shimane 690-8504, Japan

[4]RIKEN SPring-8 Center, 1-1-1, Koto, Sayo, Hyogo 679-5148, Japan

[5]Research Center for Energy and Environmental Materials (GREEN), National Institute for Materials Science (NIMS), 1-1 Namiki, Tsukuba, Ibaraki 305-0044, Japan

[6]International Center for Synchrotron Radiation Innovation Smart (SRIS), Tohoku University, 2-1-1 Katahira, Aoba-ku, Sendai, Miyagi 980-8577, Japan

[7]Department of Metallurgy, Materials Science and Materials Processing, Graduate School of Engineering, Tohoku University, 6-6-2 Aoba-yama, Aoba-ku, Sendai, Miyagi 980-8579, Japan

[8]Institute of Multidisciplinary Research for Advanced Materials (IMRAM), Tohoku University, 2-1-1 Katahira, Aoba-ku, Sendai, Miyagi 980-8577, Japan

[9]Institute for Materials Research, Tohoku University, 2-1-1 Katahira, Aoba-ku, Sendai, Miyagi 980-8577, Japan

[10]Graduate School of Chemical Sciences and Engineering, Hokkaido University, Sapporo, Hokkaido 060-0810, Japan

[11]Graduate School of Science and Technology, Gunma University, 1-5-1 Tenjin-cho, Kiryu, Gunma 376-8515, Japan

[12]National Synchrotron Radiation Research Center, Hsinchu 30076, Taiwan

[13]SR Center, Ritsumeikan University, Kusatsu, 1-1-1 Nojihigashi, Kusatsu, Shiga 525-8577, Japan

[14]Advanced Chemical Energy Research Center, Institute of Advanced Sciences, Yokohama National University, 79-5 Tokiwadai, Hodogaya-ku, Yokohama, Kanagawa 240-8501, Japan

*corresponding author, e-mail: yabuuchi-naoaki-pw@ynu.ac.jp

Supporting Figures

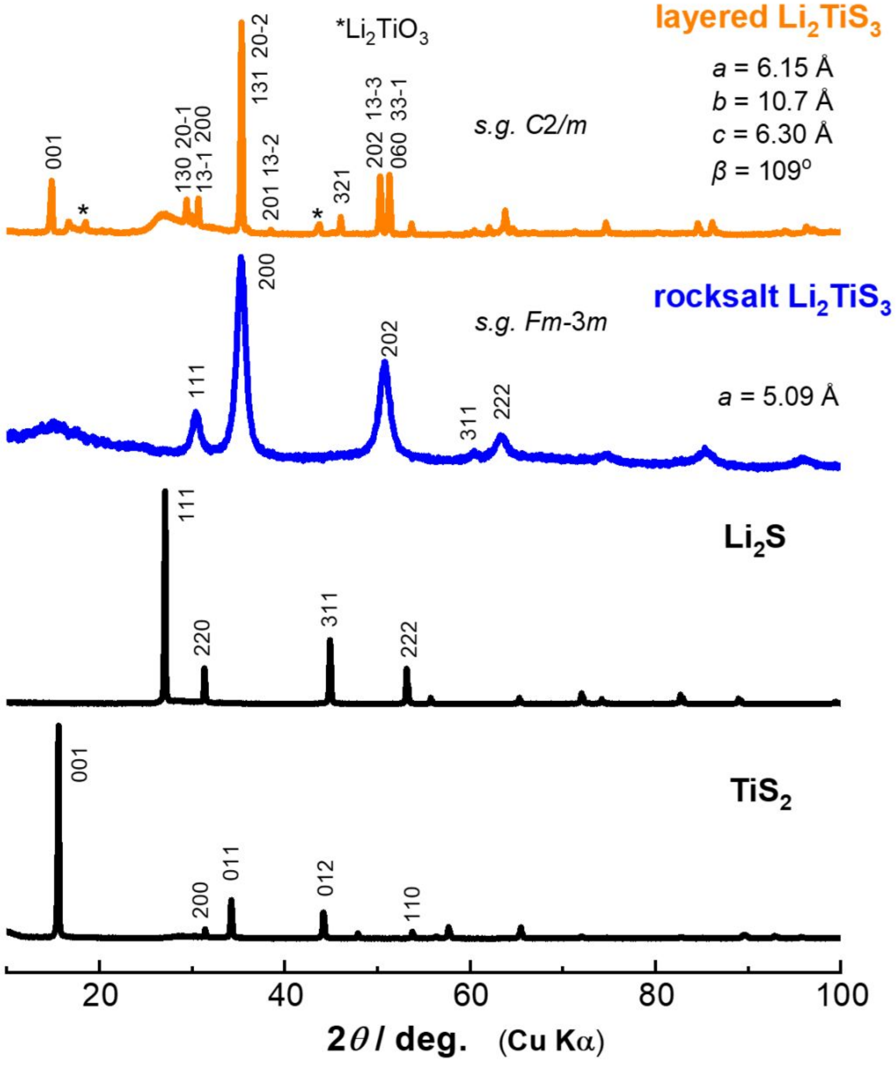

Figure S1. XRD patterns of caron-ordered layered and cation disordered rocksalt Li_2TiS_3 with the data of precursors, Li_2S and TiS_2.

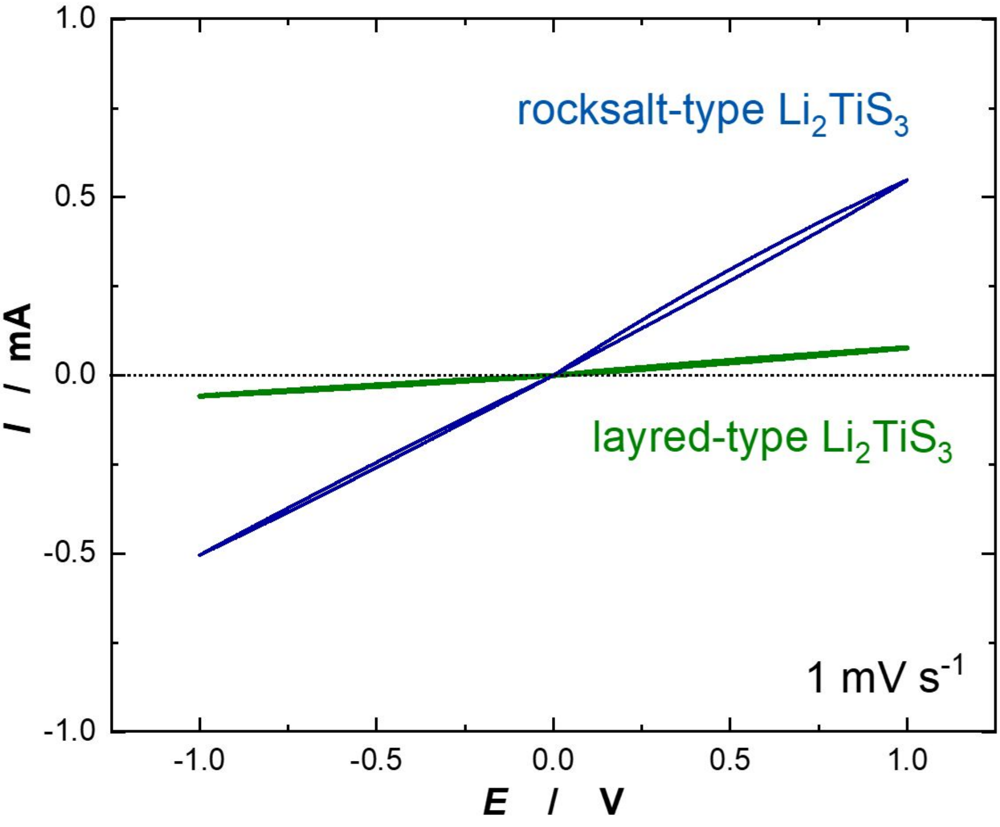

Figure S2. Cyclic voltammetry curves of powder samples of ordered (layered) and disordered (rocksalt) Li_2TiS_3.

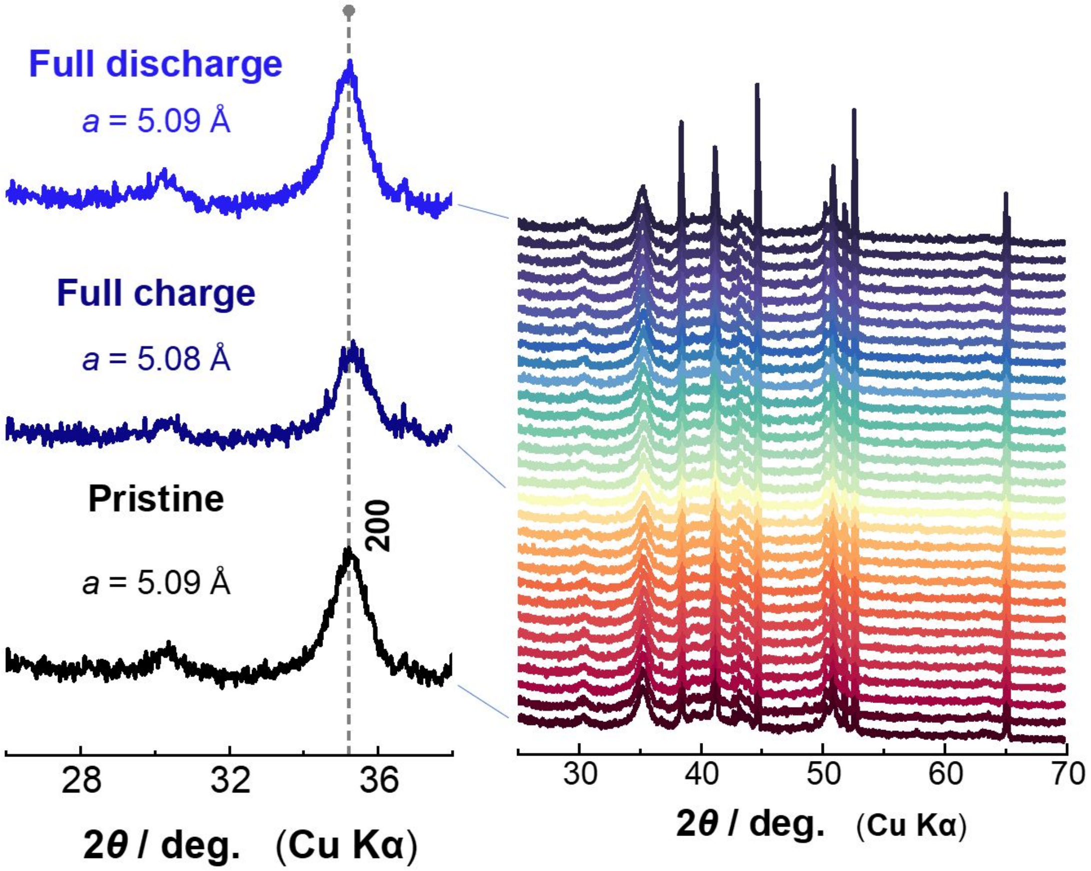

Figure S3. Original XRD data for *in-situ* XRD study of $Li_{2-x}TiS_3$

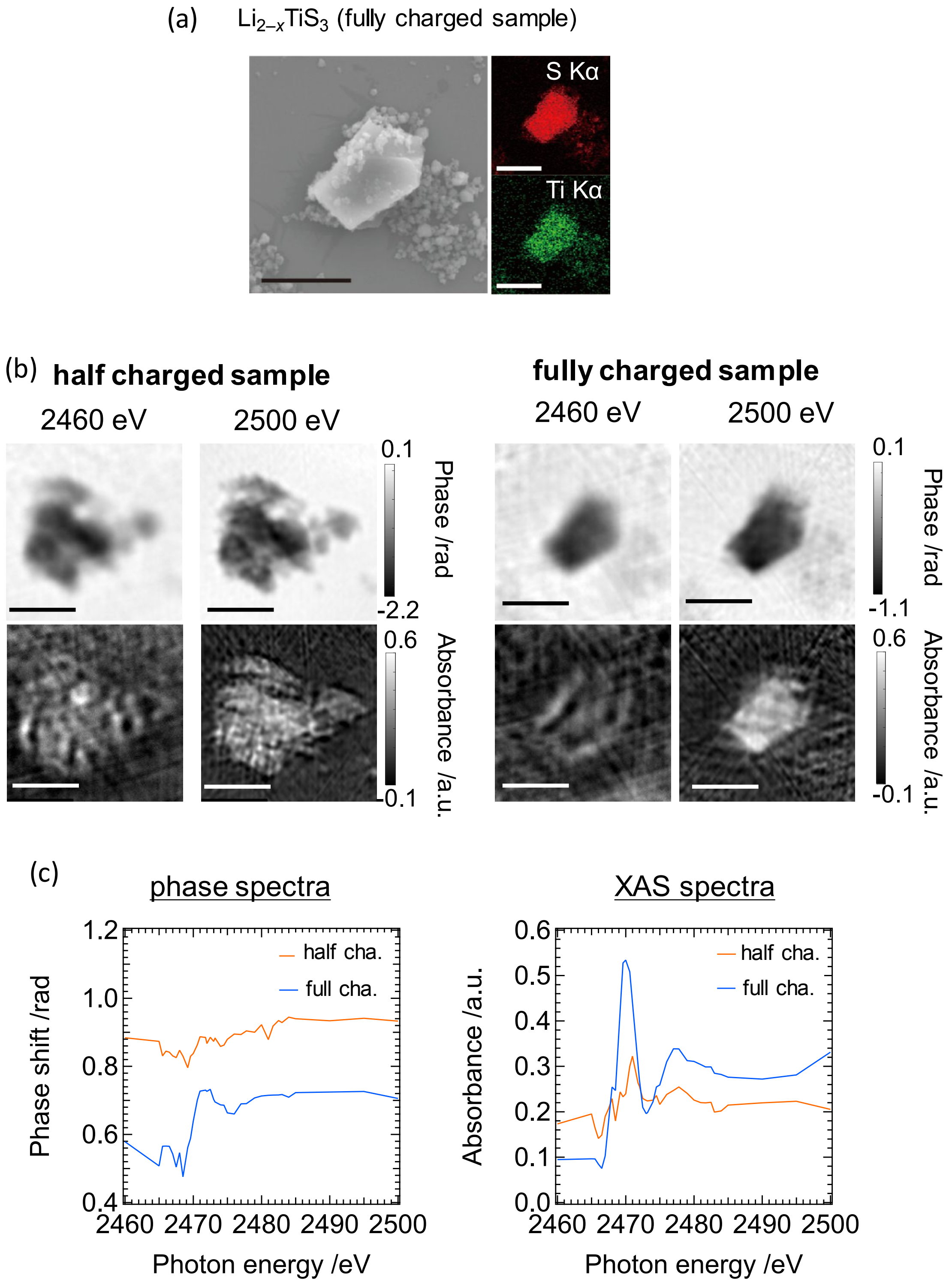

Figure S4. (a) A SEM/EDX image of fully charged $Li_{2-x}TiS_3$ with the disordered structure, (b) reconstructed phase and absorption images obtained by X-ray ptychography, and (c) phase and XAS spectra for half-charged and fully charge samples. Scale bars correspond to 2.5 μm.

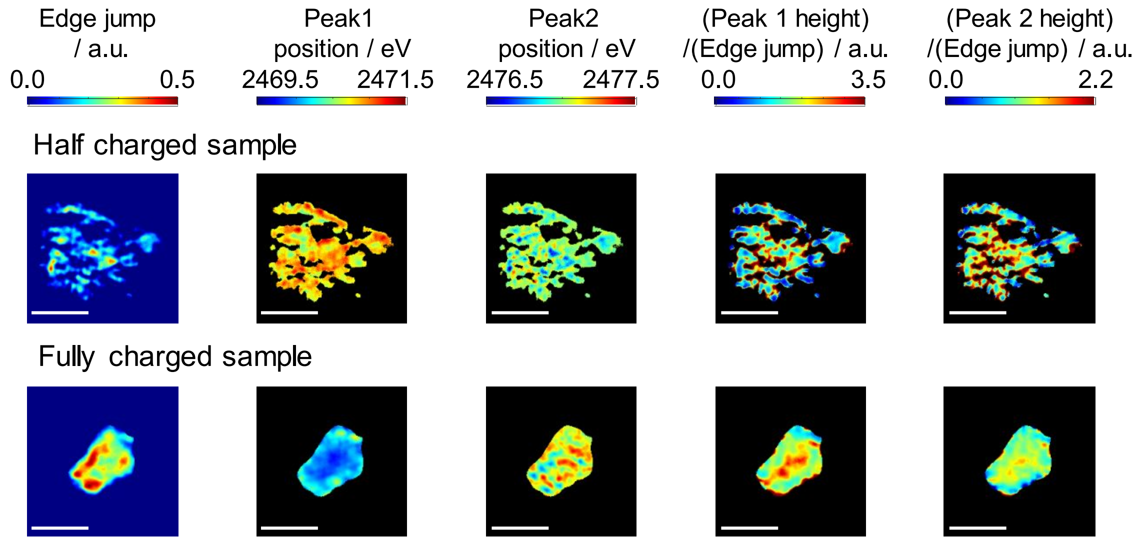

Figure S5. Chemical state maps, edge-jumps, normalized peak position and heights, obtained by X-ray spectroscopic ptychography measurements for half-charged and fully charged $Li_{2-x}TiS_3$ with the disordered structure. Scale bars correspond to 2.5 μm.

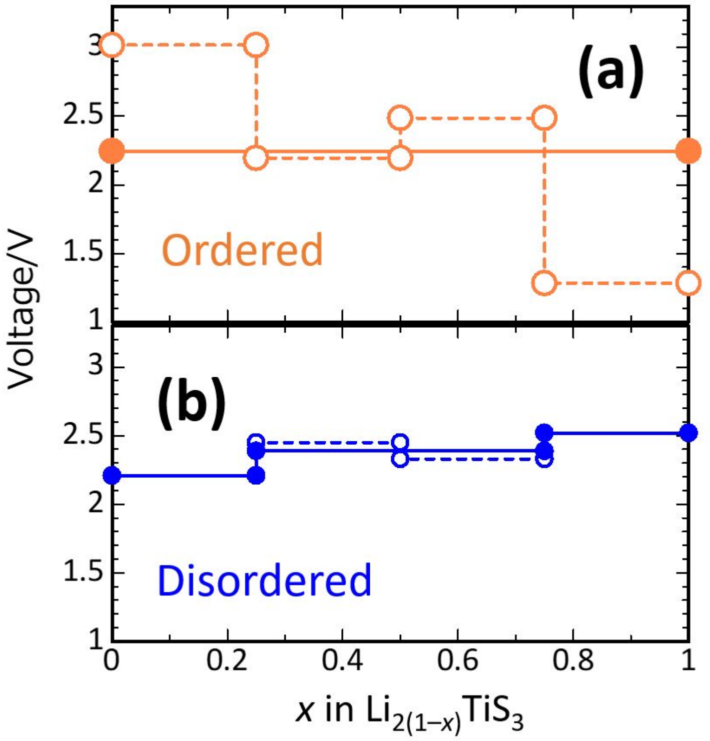

Figure S6. DFT calculated voltage profile as a function of composition x in $Li_{2(1-x)}TiS_3$. Panel (a) and (b) correspond to disordered and ordered structures, respectively. Solid lines represent thermally equilibrium reaction, where reaction path follows convex hull in **Figure 5e**. Hatched lines consider formation of metastable phases which refer the lowest energies for each composition x in $Li_{2(1-x)}TiS_3$ (x = 0, 0.25, 0.5, 0.75, and 1.0).

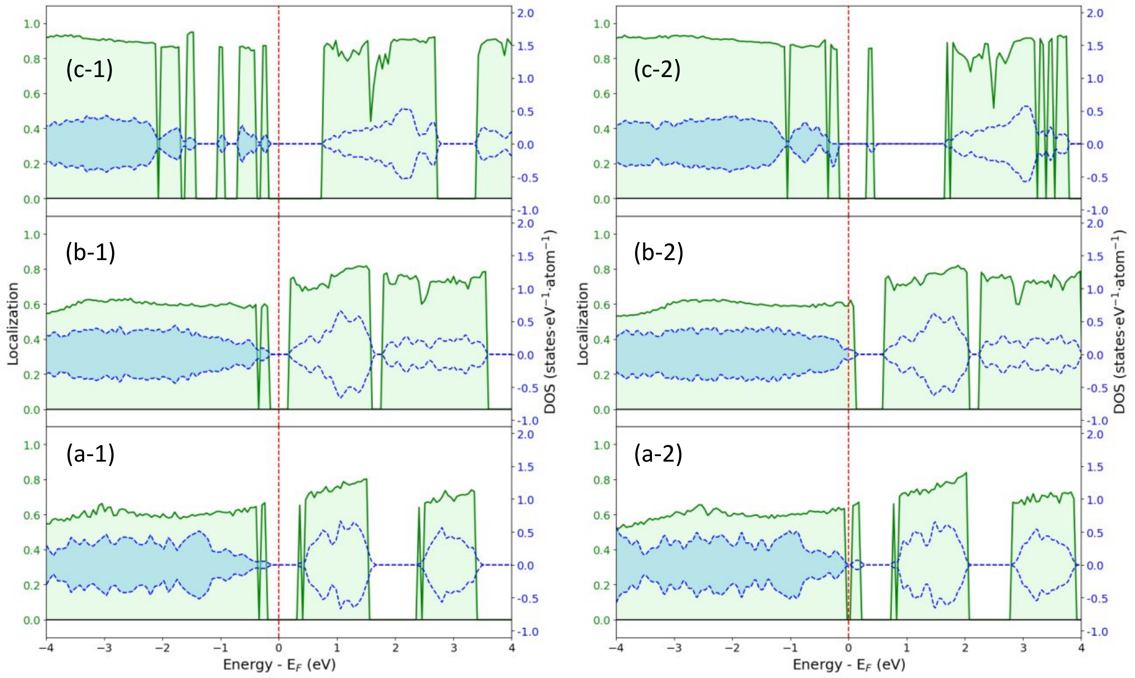

Figure S7. Electron localization factors (green solid line, left axis) and corresponding density of states (DOS, blue shaded area with dashed line, right axis) for (a) ordered Li_2TiS_3, (b) disordered Li_2TiS_3, and (c) disordered Li_2TiO_3. Panels labeled "-1" and "-2" correspond to the configurations before and after slight lithium removal, respectively. The localization represents the weight of each energy state projected onto the augmentation sphere of each atom, with higher values indicating stronger electron localization. After the partial Li extraction from disordered Li_2TiS_3, a metallic-like band is formed (b-2). Positive and negative values in the DOS correspond to spin-up and spin-down states, respectively.

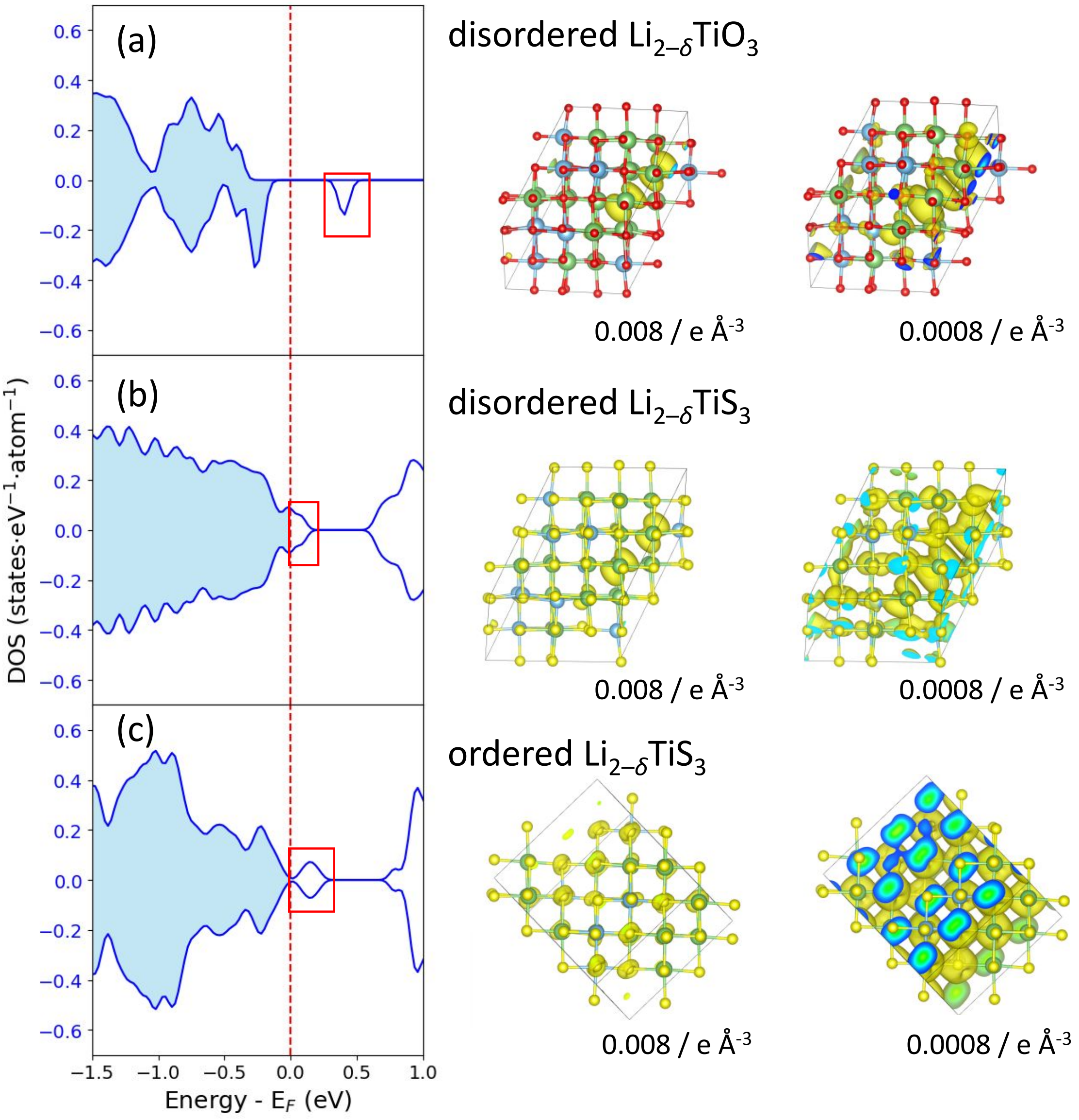

Figure S8. Magnified views of the density of states (DOS) near the Fermi level for (a) disordered $Li_{2-\delta}TiO_3$, (b) disordered $Li_{2-\delta}TiS_3$, and (c) ordered $Li_{2-\delta}TiS_3$, each after slight lithium removal. The red dashed line indicates the Fermi level. The hole states highlighted by red boxes are further visualized on the right side as isosurface projections of charge density at two isovalues: 0.008 e/Å^3 and 0.0008 e/Å^3.
